# Tuberculosis knowledge, attitude and practice among healthcare workers during the 2016 Hajj

**DOI:** 10.1371/journal.pone.0210913

**Published:** 2019-01-25

**Authors:** Badriah Alotaibi, Yara Yassin, Abdulaziz Mushi, Fuad Maashi, Abin Thomas, Gamal Mohamed, Amir Hassan, Saber Yezli

**Affiliations:** 1 The Global Centre for Mass Gatherings Medicine, Ministry of Health, Riyadh, Saudi Arabia; 2 King Abdullah International Medical Research Center, Ministry of National Guard Health Affairs, Riyadh, Kingdom of Saudi Arabia; 3 Liverpool School of Tropical Medicine, Liverpool, United Kingdom; The University of Georgia, UNITED STATES

## Abstract

**Background:**

Given the inherent characteristics of the Hajj pilgrimage, the event is a risk for tuberculosis (TB) infection. Early diagnosis and appropriate management of TB cases by knowledgeable and skilled healthcare workers (HCWs) are key in improving patients’ outcome and preventing transmission during the Hajj mass gathering and globally.

**Method:**

We conducted a cross-sectional study to assess knowledge, attitude and practice (KAP) of HCWs deployed during the 2016 Hajj regarding TB and its management using an anonymous self-administered questionnaire.

**Results:**

Data was collected from 540 HCWs from 13 hospitals. HCWs originated from 17 countries and included physicians, nurses and other non-administrative HCWs. Nearly half of HCWs declared having experience dealing with TB patients. In general, HCWs had average knowledge (mean knowledge score of 52%), above average attitude (mean attitude score of 73%) and good practice (mean practice score of 85%) regarding TB, based on our scoring system and cut-off points. Knowledge gaps were identified in relation to the definition of MDR-/XDR-TB and LTBI, smear microscopy results, length of standard TB treatment for drug-sensitive TB, 2^nd^ line anti-TB drugs, BCG vaccination, and appropriate PPE to be used with active PTB patients. Poor attitudes were found in relation to willingness to work in TB clinic/ward and to the management and treatment of TB patients. Poor practices were reported for commencing anti-TB treatment on suspected TB cases before laboratory confirmation and not increasing natural ventilation in TB patients’ rooms. Age, gender, nationality, occupation, length of work experience and experience dealing with TB patients were associated with knowledge scores. Age and occupation were associated with attitude scores while length of work experience and occupation were associated with practice scores. There was a weak but statistically significant positive correlation between score for knowledge and attitude (r_s_ = 0.11, p = 0.009) and attitude and practice (r_s_ = 0.13, p = 0.002).

**Conclusions:**

While the results of the study are encouraging, important knowledge gaps and some poor attitudes and practices regarding TB were identified among HCWs during Hajj. This calls for multifaceted interventions to improve HCWs KAP regarding TB including tailored, periodic TB education and training aimed at boosting knowledge and improving behaviour.

## Introduction

Early diagnosis and appropriate management of tuberculosis (TB) cases by knowledgeable and skilled healthcare workers (HCWs) are key in addressing this global health issue [1]. The Hajj religious mass gathering in the Kingdom of Saudi Arabia (KSA) attracts over 2 million pilgrims from around 180 different countries [2]. Many pilgrims come from TB endemic areas and worship under conditions that increase the risk of TB transmission [3]. The event has been linked to increased risk of TB infection and both diagnosed and undiagnosed TB have been reported at the pilgrimage [3, 4]. During Hajj 1000s of HCWs are deployed from across the Kingdom to ensure healthcare for pilgrims. HCWs serving during Hajj come from different nationalities; cultural, educational and occupational backgrounds; and work in different specialities. Many are deployed to temporary healthcare facilities attending large numbers of patients in a limited period of time.[5] While this workforce receives various trainings in preparation for Hajj work, including relating to infection prevention and control [6], it is unclear whether this is adequate to improve their collective knowledge, attitude and practice (KAP) regarding TB and its management during or after the event.

Knowledge gaps as well as poor attitudes and practices in relation to infection prevention and control, including TB control, have been documented among HCWs working in Hajj [5]. Numerous reports worldwide found that HCWs have important knowledge gaps in relation to TB diagnosis, treatment and infection prevention and control; have some negative attitudes and stigma connected with the disease; and engage in poor practices, all of which contribute to their increased risk of infection and negative impacts on patients and the community [7–23]. However, no study specifically examined KAP of HCWs working in Hajj in relation to TB. The current study investigated the latter with the aim of identifying knowledge gaps and poor attitudes or behaviours among HCWs that could facilitate TB transmission or impact TB management during or after the event. The results can serve as a basis for identifying difficulties, limitations and capacities for enhancement, and direct resources and efforts towards the appropriate interventions for improvement.

## Methods

### Study design, setting and population

This cross-sectional study was conducted in Mecca, Saudi Arabia, among HCWs from 13 hospitals serving pilgrims including in Hajj holy sites. The study was conducted during the 2016 Hajj season from 2^nd^ to 12^th^ September corresponding to the 1^st^ to 11^th^ of DulHija in the Islamic calendar. It is estimated that 13,000 HCWs are mobilised during Hajj. The sample size was determined using a margin of error of 5%, a confidence interval (CI) of 95%, and an expected response rate of 50% to most of the main questions. The minimum sample size estimated for the study was 374. We enrolled a larger sample size of 540 non-administrative HCWs to account for errors and non-respondents.

### Survey design and scoring system

Data was collected using an anonymous self-administered structured questionnaire developed in both English and Arabic. After introducing themselves and explaining the survey’s purpose and the objectives of the study, the research team members distributed and collected the study questionnaires from HCWs once they were filled. The survey was designed to collect KAP information concerning TB including TB microbiology and epidemiology, transmission, diagnosis, treatment and infection prevention and control. The questionnaire was developed by reviewing available questionnaires in the literature [9, 10, 17, 24] and the WHO guidelines for TB KAP surveys [25] but was tailored for the Hajj setting and the study objectives. The questionnaire was then reviewed, piloted among 20 HCWs before Hajj and validated. The Cronbach’s alpha (coefficient α) for the knowledge, attitude and practice sections of the questionnaire were 0.78, 0.72 and 0.86 respectively and were deemed acceptable.

A scoring system was developed to score the KAP responses. Incorrect/inappropriate or uncertain (don’t know) responses were given a 0 score, while 1 point was given for choosing the correct/appropriate answer; a correct/appropriate response was based on current literature and best practice. For multiple choice questions with more than one correct answer, 1 score was given for choosing the correct/appropriate response and for not choosing the incorrect/inappropriate responses. The score for the question was then divided by the total number of multiple choices in the question to standardise the result to be between 0 and 1. Scores for each section of the questionnaire (knowledge, attitude and practice) were then standardized to give overall scores ranging between 0 and 1. The overall mean scores were then further divided into 5 categories to reflect the level of KAP among HCWs. These were: poor (score 0–0.2), below average (score >0.2–0.4), average (score >0.4–0.6), above average (score >0.6–0.8) and good (score >0.8–1).

### Statistical analysis

Descriptive statistics such as mean, standard deviation (SD), median, and interquartile range (IQR) were computed for quantitative variables and frequencies and percentages were calculated for categorical variables. Cronbach’s α, was used to measure reliability and internal consistency for the KAP questions. The difference of KAP score with respect to individual covariates was evaluated by the Mann–Whitney U test or Kruskal-Wallis test as appropriate. Simultaneous effects of covariates towards knowledge, attitude, and practice scores were assessed by fitting quantile regression models. Regression models were fitted for 25^th^, 50^th^, and 75^th^ percentiles of the scores to evaluate the relationship between covariates and KAP scores. The covariates were selected by assessing their relationship towards scores in bivariate analysis. From the independent variable list, “education level” was removed while model fitting, because it was highly associated with “occupation” variable and including occupation in the model, showed a better fit. Correlation between knowledge attitude and practice was examined using the Spearman correlation coefficient. All of the tests for significance were two-sided and p values < 0.05 were considered statistically significant. All analyses were done using SPSS 22.0 (SPSS Inc., Chicago, USA) and SAS 9.4 (SAS Institute Inc., NC, USA) software program.

### Ethics and confidentiality

All study participants were briefed about the study and gave verbal consent before enrollment. The study was approved by the King Fahad Medical City Ethics Committee and the Institutional Review Board (IRB log: 16-329E) and conducted in accordance with the Ethics Committee’s guidelines. The HCWs KAP survey forms were anonymous and did not include any identifiers or personal information of the participants.

## Results

### Characteristics of the study population

Characteristics of the study population are summarized in Table 1. Data was collected from 540 HCWs from 13 hospitals serving pilgrims during Hajj. HCWs represented 17 nationalities but over half (52.1%) were Saudi nationals. Among the non-Saudi nationals, the highest number of HCWs were from the Philippines (63, 27.9%), Egypt (53, 23.5%) and India (31, 13.7%). HCWs had a mean age of 35.1 (SD = 8.92) years ranging from 22–64 years and a male:female ratio of 1:1. Most HCWs (428, 81%) had a Bachelor degree or a diploma (lower than a bachelor degree) and over half (51.8%) were nurses. Among the 102 HCWs who reported their occupation as “other”, 48 (47.1%) were laboratory/diagnostic staff, and 35 (34.3%) were pharmacists. The main wards the HCWs were assigned to in non-Hajj time were ICUs (76, 14.5%), ERs (110, 21%) and general medical wards (91, 17.3%). These same wards (ICUs, ERs and general medical wards) were also the most represented during Hajj deployment (14%, 28.8% and 21.6% respectively). Few HCWs (30, 5.8%) worked <1 year in their current position. Nearly half of the participants declared dealing with TB patients during their non-Hajj work, and 18% reported that they dealt with TB patients during Hajj specifically.

**Table 1:** Characteristics of the study population.

Characteristics	Category	Freq	%
**Age**	<30	158	34.10
	30–39	190	40.90
	40–49	71	15.30
	>49	45	9.70
**Gender**	Male	258	48.22
**Nationality**	Saudi	246	52.10
	Non-Saudi	226	47.90
**Education Level**	Other	8	1.51
	Diploma	191	36.11
	Bachelor	237	44.80
	Master	53	10.02
	Doctorate/Speciality certificate	40	7.56
**Occupation**	Other	102	19.21
	Physician	154	29.00
	Nurse	275	51.79
**Current position length**	<1 year	30	5.76
	1–5 years	177	33.97
	>5–10 years	169	32.44
	>10 years	145	27.83
**Dealing with TB patients**	During non-Hajj work	261	51.18
	During Hajj deployment	85	18.12

TB; tuberculosis

### Tuberculosis knowledge among healthcare workers

The mean scores for each knowledge question are presented in Table 2. According to our scale, HCWs had average TB knowledge (overall mean knowledge score = 0.52, SD = 0.17) and most HCWs (78%) had average or above, knowledge score (mean knowledge score >0.4%). However important knowledge gaps were noted. For example, 21% of HCWs thought TB is caused by a virus, 18% indicated that surgical masks are appropriate PPE to use when dealing with patients with active TB and (17–42%) thought TB is transmitted via contaminated surfaces, sharing food or drinks, kissing or shaking hands. Also, 43–60% of HCWs did not recognise some common symptoms of TB such as tiredness/fatigue, chest pain, loss of appetite and night sweats.

**Table 2:** Mean knowledge scores for HCWs regarding TB.

Knowledge question	N	Min	Max	Mean	SD
1. TB falls under which type of infection	485	0	1	0.75	0.43
2. PTB main symptoms	520	0.40	1	0.76	0.14
3. PTB diagnostic tests	519	0.27	1	0.68	0.13
4. PTB transmission	540	0.29	1	0.71	0.15
5. Latent TB screening tests	487	0.27	1	0.74	0.11
6. Percentage of active PTB positive for TB on smear microscopy	540	0	1	0.16	0.37
7. Most useful sputum collection method for PTB diagnosis	435	0	1	0.87	0.33
8. 1^st^-line anti-TB drug	381	0.40	1	0.85	0.10
9. 2^nd^-line anti-TB drug	308	0.10	1	0.50	0.24
10. Definition of MDR-TB	501	0	1	0.25	0.43
11. Definition of XDR-TB	479	0	1	0.13	0.33
12. Length of standard treatment of drug-sensitive TB	513	0	1	0.31	0.46
13. HIV patients are more vulnerable to contracting TB	519	0	1	0.81	0.39
14. PTB is curable	517	0	1	0.88	0.32
15. MDR-TB is curable	504	0	1	0.51	0.5
16. BCG vaccination is protective against TB	514	0	1	0.36	0.48
17. PTB is transmitted via the airborne route	507	0	1	0.84	0.37
18. Patients with LTBI can spread the disease	515	0	1	0.33	0.47
19. Patients with LTBI have positive reaction on TST/IGRA tests	505	0	1	0.57	0.49
20. Patient with TB become non-infectious soon after initiating appropriate treatment	512	0	1	0.41	0.49
21. Only patients with active TB can spread the disease	511	0	1	0.60	0.49
22. Appropriate PPE to use with active PTB patients	514	0	1	0.82	0.38
23.Using PPE to protect HCWs from TB is important	522	0	1	0.90	0.30
**Overall score**	**308**	**0.02**	**0.90**	**0.52**	**0.17**

TB; tuberculosis, PTB; pulmonary tuberculosis, LTBI; latent TB infection, HIV; human immunodeficiency virus, PPE; personal protective equipment, HCW; healthcare worker, MDR; multidrug-resistant, XDR; extensively drug-resistant, TST: tuberculin skin tests (TST), AFN; acid-fast bacilli, IGRA; interferon gamma release assay, CBC; complete blood count, BCG; Bacillus Calmette-Guérin. Min; minimum, Max, maximum, SD; standard deviation, N; number of observations

Deficiencies in knowledge of TB screening/diagnostic tests were also identified. For instance, 71% and 66% of HCWs identified chest X-ray and TST respectively as diagnostic tests for pulmonary TB and only 12% recognised that GeneXpert MTB/RIF was a diagnostic tool. Also, 35% and 44% of HCWs identified sputum acid-fast bacilli smear test and sputum culture respectively as screening tests for Latent TB infection (LTBI) but only 13% thought IGRA was a screening tests for LTBI. Only 25% of HCWs knew the definition of MDR-TB and even fewer (13%) knew XDR-TB. Inadequate or poor knowledge was also recorded for questions relating to the length of standard treatment for drug-sensitive TB, infectiousness of people with LTBI, the results of smear microscopy, whether BCG vaccination was protective against TB, 2^nd^-line anti-TB drugs and whether MDR-TB was curable (S1 Table). Only 25% of HCWs (mostly nurses) reported attending a lecture/seminar/workshop on TB in the previous 12 month.

### Attitude of healthcare workers towards tuberculosis

The mean scores for each attitude question are presented in Table 3. The attitude of HCWs towards TB was deemed above average (overall mean attitude score = 0.73, SD = 0.15) with 84.7% having above average or higher attitude score (mean attitude score >0.6%). Most HCWs were willing to be educated on TB (95%) and teach others (>85%), willing to be screened for TB if they had suggestive symptoms (94%), would use PPE when dealing with pulmonary TB patients even when it is uncomfortable (82%), would not suspend TB treatment if the patient was feeling better (87%) and trust diagnostic results from laboratory (92%). Also, most would not resign if they were posted to a TB clinic/ward (84%) and they had no issues examining/treating a TB patient (85%). However, some poor attitudes were identified. Nearly 60% of HCWs were not willing to work in TB clinic/ward, 32% did not think that all TB patients should be isolated and 39% would start TB treatment for a suspected TB case before diagnosis is confirmed if the patient was very ill. While 30% of HCWs thought they had a very low risk of acquiring TB from their patients, 69% were worried about nosocomial acquisition of TB.

**Table 3:** Mean attitude scores for HCWs regarding TB.

Attitude statement /question	N	Min	Max	Mean	SD
1. Would you be willing to work in a TB clinic/ward?	521	0	1	0.41	0.49
2. Would you resign from work if you are posted to a TB clinic/ward?	515	0	1	0.84	0.37
3. Would you be willing to be screened for TB if you had suggestive symptoms?	512	0	1	0.94	0.23
4. Do you think all TB patients should be isolated for treatment?	527	0	1	0.66	0.47
5. It is ok to allow a TB patient to leave the hospital soon after initiating appropriate treatment?	521	0	1	0.53	0.50
6. Would you be willing to attend seminars on TB?	512	0	1	0.95	0.22
7. Would you recommend the suspension of treatment if a TB patient is feeling better?	518	0	1	0.87	0.34
8. Would you start TB treatment for a TB patient before diagnosis is confirmed if a suspected TB patient is very ill?	515	0	1	0.61	0.49
9. Would you use a face mask when dealing with a PTB patient even when it is uncomfortable?	521	0	1	0.82	0.38
10. Would you trust the result the laboratory provides you on sputum cultures?	524	0	1	0.91	0.29
11. I would not accept to examine/treat TB patients	510	0	1	0.85	0.35
**Overall score**	**510**	**0**	**1**	**0.73**	**0.15**

TB; tuberculosis, PTB; pulmonary tuberculosis, Min; minimum, Max, maximum, SD; standard deviation, N; number of observations

### Tuberculosis practice among healthcare workers

The mean scores for each practice question are presented in Table 4. HCWs reported good practice regarding TB management and infection prevention (overall mean practice score = 0.81, SD = 0.17). Most reported performing hand hygiene, using appropriate PPE when dealing with PTB patients or handling TB samples (>83%), not using soiled N95 respirators (88%), isolating TB cases from other patients (92%), and separating TB patients from those with HIV (91%). Over 90% of HCWs reported requesting sputum tests when they suspect active TB and ensuring the samples’ quality. Also, 74% order HIV test when a patient is diagnosed with TB and 90% request liver function tests before starting anti-TB treatment. In addition, 88% of HCWs reported requesting contact tracing for all confirmed TB cases and 79% start those contacts who are positive for IGRA/TST on isoniazid/rifampicin prophylaxis. Poor practice was reported for commencing anti-TB treatment on suspected TB cases before laboratory confirmation (33.5%) and not opening the windows in TB patients’ rooms to increase natural ventilation (51%).

**Table 4:** Mean practice scores for HCWs regarding TB.

Practice statement	N	Min	Max	Mean	SD
1. I usually perform hand hygiene and wear PPE before contact with TB patients/samples	519	0	1	0.92	0.27
2. I usually wear N95 respirator when caring for patient with PTB/working on TB samples	514	0	1	0.83	0.37
3. I request sputum tests when I suspect active TB	516	0	1	0.93	0.25
4. Always put the patient with active TB in the isolated room	511	0	1	0.93	0.26
5. I open windows when possible in TB patients’ rooms to increase natural ventilation	511	0	1	0.49	0.50
6. I order HIV test when I diagnose active TB	513	0	1	0.74	0.44
7. Always put the patient with known TB separated from HIV patients	522	0	1	0.91	0.28
8. Sometimes I use wet or soiled N95 respirator	519	0	1	0.88	0.32
9. I always make sure that samples are sputum and not saliva before sending them to the lab/before testing in the lab	519	0	1	0.90	0.30
10. I commence anti-TB drugs on suspect TB cases before lab confirmation	522	0	1	0.67	0.47
11. I request contact tracing for all confirmed TB cases	515	0	1	0.88	0.33
12. I request liver function tests before starting anti-TB treatment	523	0	1	0.91	0.29
13. I start contacts of active TB cases who are positive for IGRA/TST tests on INH/RIF prophylaxis	516	0	1	0.79	0.41
**Overall score**	**511**	**0**	**1**	**0.81**	**0.17**

TB; tuberculosis, PTB; pulmonary tuberculosis, HIV; human immunodeficiency virus, PPE; personal protective equipment, TST: tuberculin skin tests (TST), IGRA; interferon gamma release assay, INH; isoniazid, RIF; rifampicin, Min; minimum, Max, maximum, SD; standard deviation, N; number of observations

### Demographic variables and KAP scores

There was a statistically significant difference in knowledge scores in relation to age groups, gender, nationality, level of education, occupation and length of work experience. HCWs in the older age groups, females, non-Saudis, physicians, those with higher level of education and longer work experience had higher mean knowledge scores. There was no statistically significant difference in the variables and attitude scores and only level of education showed significant difference in relation to practice scores (Table 5).

**Table 5:** Demographic variables and KAP scores of HCWs.

Variable		Knowledge scores				Attitude scores				Practice scores			
		N	Mean	SD	P-value*	N	Mean	SD	P-value*	N	Mean	SD	P-value*
**Age**					**<0.0001**				0.8501				0.6622
	<30	158	0.48	0.16		158	0.73	0.15		158	0.79	0.17	
	30–39	190	0.51	0.17		190	0.73	0.16		190	0.80	0.19	
	40–49	71	0.62	0.13		71	0.73	0.15		71	0.80	0.16	
	>49	45	0.58	0.13		45	0.72	0.13		45	0.83	0.10	
**Gender**					**0.0461**				0.0667				0.1404
	Female	277	0.53	0.14		277	0.74	0.13		277	0.81	0.18	
	Male	258	0.50	0.18		258	0.71	0.16		258	0.80	0.20	
**Nationality**					**<0.0001**				0.1623				0.3412
	Saudi	246	0.44	0.15		246	0.72	0.16		246	0.79	0.20	
	Non-Saudi	226	0.61	0.12		226	0.75	0.14		226	0.80	0.13	
**Education level**					**<0.0001**				0.5845				**0.0491**
	Diploma	191	0.43	0.15		191	0.72	0.16		191	0.81	0.19	
	Bachelor	237	0.55	0.15		237	0.74	0.15		237	0.79	0.15	
	Master	53	0.59	0.11		53	0.72	0.12		53	0.82	0.16	
	Doctorate/Speciality certificate	40	0.62	0.16		40	0.74	0.12		40	0.86	0.10	
**Occupation**					**<0.0001**				0.5728				0.1663
	Other	102	0.40	0.15		102	0.72	0.18		102	0.80	0.23	0.1663
	Physician	154	0.59	0.16		154	0.72	0.13		154	0.81	0.17	
	Nurse	275	0.51	0.15		275	0.74	0.15		275	0.80	0.14	
**Current position length**					**0.0029**				0.5606				0.0711
	<1 yr	30	0.50	0.16		30	0.71	0.17		30	0.72	0.27	
	1–5 yr	177	0.50	0.15		177	0.74	0.14		177	0.79	0.16	
	>5–10 yr	169	0.51	0.17		169	0.73	0.16		169	0.82	0.15	
	>10 yr	145	0.56	0.15		145	0.73	0.13		145	0.83	0.14	
**Non-Hajj TB patient contact**					0.0749		0.72	0.16	0.1914				0.3053
	No	249	0.50	0.17		249	0.74	0.13		249	0.81	0.17	
	Yes	261	0.54	0.15		261				261	0.80	0.16	
**Attended a TB seminar in the previous year**					0.1942		0.73	0.15	0.8862				0.1473
	No	394	0.51	0.17		394	0.73	0.14		394	0.80	0.17	
	Yes	129	0.53	0.14		129	0.73	0.18		129	0.82	0.17	

*p-value for the Mann–Whitney U or Kruskal-Wallis test

N; number of observations, SD; standard deviation, TB; tuberculosis, yr; years

Quantile regression results indicate that a number of variables were associated with KAP scores. The direction and the significance of the association of the variables with the KAP scores differed across the 25^th^, 50^th^ and 75^th^ KAP score quantiles (Table 6). Nationality, occupation and length of work experience were significantly associated with knowledge scores at the three different quantiles. In general, non-Saudis had better knowledge than Saudis, physicians were more knowledgeable than both nurses and other HCWs, while HCWs with <1 year in their current position were less knowledgeable than those who had longer experience. Gender was associated with knowledge scores at the 25^th^ and 50^th^ quantiles only, with females being more knowledgeable than males. Similarly, age and experience dealing with TB patients were significantly associated with knowledge scores at the 50^th^ and 25^th^ quantiles respectively.

**Table 6:** Summary of the association between variables and KAP scores of HCWs at the 25^th^, 50^th^ and 75^th^ quantiles.

Variable		Coef for knowledge scores			Coef for attitude scores			Coef for practice scores		
		(SE)			(SE)			(SE)		
		25^th^	50^th^	75^th^	25^th^	50^th^	75^th^	25^th^	50^th^	75^th^
**Intercept**		**0.363**	**0.4413**	**0.5711**	**0.5758**	**0.6862**	**0.7596**	**0.5974**	**0.7778**	**0.8594**
		(0.050)	(0.041)	(0.060)	(0.059)	(0.050)	(0.051)	(0.087)	(0.052)	(0.045)
**Age**										
	<30	0.0069	0.0017	0.0125	0.000	-0.016	0.000	-0.0037	0.000	-0.0002
		(0.045)	(0.029)	(0.033)	(0.041)	(0.037)	(0.039)	(0.058)	(0.033)	(0.023)
	30–39	0.0192	0.0116	0.0277	0.0303	0.0225	**0.0707**	0.0067	0.000	0.0064
		(0.038)	(0.041)	(0.029)	(0.038)	(0.032)	(0.033)	(0.053)	(0.030)	(0.021)
	40–49	0.0708	**0.0699**	0.0383	0.000	0.0216	0.000	0.0009	0.000	0.000
		(0.034)	(0.020)	(0.033)	(0.042)	(0.028)	(0.023)	(0.045)	(0.029)	(0.018)
**Gender**										
	Female	**0.0452**	**0.0381**	0.0277	0.0333	0.0219	0.000	0.0067	0.000	0.0002
		(0.017)	(0.018)	(0.024)	(0.026)	(0.023)	(0.019)	(0.034)	(0.022)	(0.015)
**Nationality**										
	Non-Saudi	**0.145**	**0.1065**	**0.0804**	0.0303	0.0352	0.0202	0.0143*	0.000*	-0.0066*
		(0.018)	(0.014)	(0.018)	(0.022)	(0.020)	(0.019)	(0.029)	(0.018)	(0.016)
**Occupation**										
	Other	**-0.1112**	**-0.1056**	**-0.1585**	0.000	**0.0684**	0.0202	-0.0187*	0.0128*	0.0639*
		(0.036)	(0.029)	(0.033)	(0.038)	(0.030)	(0.027)	(0.052)	(0.032)	(0.020)
	Nurse	-0.039	**-0.0455**	**-0.0977**	0.0273	0.0298	0.027)	-0.025	0.000	-0.0066
		(0.023)	(0.020)	(0.027)	(0.030)	(0.024)	(0.020)	(0.039)	(0.022)	(0.018)
**Current position length**										
	1–5 years	0.0388	0.0591	0.0601	0.0606	0.0578	0.0384	0.1067	0.0684	0.0629
		(0.033)	(0.033)	(0.032)	(0.048)	(0.037)	(0.030)	(0.068)	(0.042)	(0.035)
	>5–10 years	0.0397	0.066	0.0653	0.0303	0.0054	0.0384	0.117	0.0556	0.0639
		(0.037)	(0.032)	(0.035)	(0.051)	(0.040)	(0.034)	(0.069)	(0.044)	(0.035)
	>10 years	**0.0764**	**0.0756**	**0.0891**	0.0606	0.0058	0.0384	**0.1567**	0.0684	0.0639
		(0.037)	(0.033)	(0.044)	(0.052)	(0.040)	(0.038)	(0.075)	(0.044)	(0.035)
**Non-Hajj TB patient contact**										
	No	**-0.0616**	-0.0146	-0.0135	-0.0303	0.000	0.000	0.0183	0.000	0.0064
		(0.018)	(0.013)	(0.015)	(0.020)	(0.018)	(0.011)	(0.022)	(0.014)	(0.010)

Coef; regression coefficient, SE; standard error, TB; tuberculosis

*change in direction of association across the three different quantiles

Coefficient in bold indicates statistically significant p-value associated with the coefficient

Attitude scores were significantly associated with age at the 75^th^ quantile and occupation at the 50^th^ quantile. At the latter quantiles, HCWs in the 30–39 years old age group had significantly better attitude than those >49 years old while “other” HCWs had significantly better attitude compared to physicians. Length of work experience and occupation were significantly associated with practice scores at the 25^th^ and 75^Th^ quantiles respectively. At these quantiles, “other” HCWs had significantly better practice than physicians, while HCWs with <1 year work experience had worst practice compared to those with >10 years work experience. There was a weak but statistically significant positive correlation between knowledge and attitude (r_s_ = 0.11, p = 0.009) and attitude and practice (r_s_ = 0.13, p = 0.002).

## Discussion

Knowledge deficit regarding TB among HCWs may result in substandard care, ineffective service provision, inefficient resource use, and impact on health outcomes as well as increasing the risk of TB transmission and development of resistance. In addition, the knowledge and attitude of HCWs towards TB will determine the type and quality of information passed on to the patients during health education. Inadequate or incomplete information passed on to the patients create wrong perceptions in them or strengthen the patients’ negative perceptions which are often times based on cultural beliefs and misconceptions [26, 27]. These negative perceptions are mostly inimical to appropriate health seeking behaviour, hence reducing the likelihood of completing treatment and achieving cure and increasing the risk of spread of the disease [26]. As such, the knowledge of health professionals related to TB affects not only themselves and their individual patients, but also the global population as a whole. A number of studies from around the world investigated knowledge of HCWs regarding TB and found varying results [7]. Some reported poor knowledge [13, 28], others found adequate [17, 29–31] or good knowledge [14, 32]. For example studies among HCWs from Peru [29], Mozambique [13], Russia [28], Ethiopia [33] and Lesotho [10] reported average TB knowledge scores of 51.7–74%, which are close to what we found among HCWs working in Hajj (52%). However, while we deemed TB knowledge in the current study to be average, a number of these studies reported that their scores were poor, unsatisfactory or low given their higher score cut off points for good knowledge [10, 13, 28].

Regardless of the level of TB knowledge among HCWs, knowledge gaps have been reported in most studies [7], including in ours. For instance, a quarter of HCWs we surveyed did not identify TB as a bacterial infection and nearly 12% did not know that TB is curable. These were slightly higher rates than those reported from other countries such as Iraq, Lesotho, Russia, Peru, and Vietnam, where 1–12.6% of HCWs did not know that TB is caused by a bacterium and 2.3–8% did not know that TB is curable [14, 17, 28, 29, 34]. We identified a number of misconceptions regarding the route of TB transmission which have also been reported among HCWs in other studies, albeit to a lesser extent [14, 30]. As noted by others [17], we found HCWs were unclear on the value of BCG vaccination in relation to TB prevention.

A concerning result from our study is the lack of knowledge of HCWs regarding drug-resistant TB. Evidence from several studies worldwide have found that HCWs do not always have sufficient knowledge of MDR-TB, even those working in TB facilities [10, 28, 29, 35]. Studies among TB HCWs found that only 37.2% knew the definition of MDR-TB [29], and only 59% recognised it was curable [28]. Although patients harbouring MDR and XDR strains present a formidable challenge for treatment, cure is often possible with early identification of resistance and use of a properly designed treatment regimen [1]. Hence, it is important that HCWs are aware of MDR-TB, able to diagnose it early, and are aware of the fact that patients with MDR- and XDR-TB can be cured and communicate this information to patients and the community at large.

While most HCWs recognised some common symptoms of pulmonary TB, many missed other common symptoms, including physicians. These results are similar to those reported among HCWs from Lesotho [17], Nepal [31], Vietnam [34], Brazil [36], and Iraq [14]. Lack of knowledge among HCWs regarding symptoms of TB may translate into delays in identifying, isolating and treating patients with TB or in TB patients not being diagnosed with the disease and consequent transmission in hospitals and the community. As with other reports [14, 17, 21, 29, 30, 34], we also found deficiencies in knowledge and confusion among HCWs regarding tests used to screen or diagnose active disease and those used to screen for latent infection. This appears not only to be related to lack of knowledge of the tests, especially modern techniques, but also to a confusion between active TB disease and LTBI. This confusion has been reported in other studies. For example, only 54.7% of HCWs in Nepal knew the difference between TB infection and disease [31], and 42% of HCWs from Uganda thought there was no difference between the two [30].

A striking observation in our results was the poor knowledge among HCWs regarding the use of GeneXpert MTB/RIF for TB diagnosis. It is clear that despite the WHO issuing recommendations endorsing this technology in 2010, and the widespread uptake of its use worldwide since [37], knowledge of its existence and purpose remains low in our study as well as in others [13]. It is worth noting that GeneXpert MTB/RIF is available in the Kingdom, hence lack of knowledge among HCWs may suggest that the roll out of this technology might not have been accompanied with sufficient education for HCWs [13]. The use of rapid molecular diagnostic techniques for TB such as GeneXpert MTB/RIF is important in the context of Hajj given the characteristics of the pilgrims’ population, the crowded environment, the short duration of the mass gathering, and potential for international transmission.

We also identified key knowledge gaps regarding TB treatment among HCWs in our study. In general, available data from around the world indicate a lack of knowledge of national and international TB treatment guidelines among HCWs and inappropriate knowledge of treatment regimens and treatment duration were identified in 8–100% and 5–99% of HCWs respectively [12]. Data from this study and others indicate that inappropriate treatment of TB may be a problem worldwide [38].

HCW’s attitudes towards TB patients are important factors in improving patient’s health‑seeking behaviour, compliance with prescribed treatment regimen and treatment outcome as well as preventing the development of drug-resistance [26, 35]. Our data suggest that HCWs in general had positive attitudes toward working with patients with TB, although some negative attitudes were noted. These were mostly related to willingness to work in TB clinic/ward and to some aspects of management and treatment of TB patients. We also found that most HCWs worried about nosocomial acquisition of TB even though many thought they had very low risk of acquiring TB from their patients, which is in accordance with other reports [20, 30]. Suboptimal knowledge regarding TB, its transmission and treatment, HCWs own experiences, the stigma attached to the disease and worry about TB infection have been shown to affect HCWs attitudes [24, 26, 27, 39]. These may explain why 60% of the HCWs in our study were not willing to work in TB clinic/ward.

We found that HCWs had generally good self-reported practice in relation to TB and its management. The overall mean practice score was higher than that for knowledge or attitude and was higher than data reported from other studies [13, 33]. Over 62% of the HCWs were deemed to have good overall practice scores, with only 1% receiving a poor score. While this finding is encouraging, there were a number of poor practices identified among HCWs in this study, which is consistent with previously reported work [7, 14, 23]. These poor practices dealt primarily with commencing anti-TB treatment on suspected TB cases before laboratory confirmation and not opening the windows in patients’ rooms to increase natural ventilation. The latter was probably, in part, related to the hot weather conditions in KSA, which may discourage HCWs from the practice. Weather conditions have been reported by others as barriers for opening windows by HCWs to increase natural ventilation in rooms of TB patients [17].

A number of factors were associated with better knowledge of TB among HCWs in our study including, age, gender, occupation, length of work experience and experience with TB patients. Studies from around the world reported similar factors influencing TB knowledge among HCWs [10, 13, 14, 23, 28–33]. In relation to attitude, occupation and age were significantly associated with attitude scores. In Peru, HCW’s TB attitude scores were reported to differ among different employment groups[29] while a study from Thailand found that age of HCWs was significantly linked to their attitude towards TB [32]. Practice scores were significantly different according to level of education, also, length of work experience and occupation were significant factors associated with practice scores. A study from Mozambique found significant association between TB practice scores among HCWs and level of education, occupation and experience with TB patients [13]. Demissie Gizaw et al.[33] reported that independent predictors for having good TB infection control practice among HCWs in Ethiopia, were TB work experience, TB training and level of education. We also reported a statistically significant positive correlation between knowledge and attitude and between attitude and practice. Yet, the association was weak and there was no significant association between knowledge and practice scores. Reports in the literature support the notion that there is no simple relationship between the level of knowledge, attitude and behaviours regarding TB and its management among HCWs [9, 14, 19, 33].

Given the context of Hajj, the potential global health consequences of mismanagement of TB cases at the event, and the results of the current study, interventions to improve HCWs’ KAP regarding TB are needed. HCWs should receive tailored, high quality, periodic TB education and training including before deployment to Hajj with ongoing monitoring and evaluation of the impact. Training should be determined according to job categories, needs, and educational backgrounds. Special attention should be given to training non-clinical, auxiliary and support staff to improve their KAP and prepare them to safely work in high risk settings such as TB care [28, 30, 31]. Training should not only emphasise the theoretical aspects of training but also skill-based components to impact on practice. Various means shown effective in bringing about behavioural change among HCWs should be used including traditional and non-traditional methods of communicating information. These include audit and feedback, distribution of education materials, educational meetings, local opinion leaders, outreach visits, reminders, small group discussions and simulated cases to teach management and role-plays to teach methods of evaluation of case-finding and treatment [23, 40]. Educational and training activities alone or in combination with other intervention were previously reported to have improved HCWs’ KAP regarding TB and reduced nosocomial transmission of the disease [41–43].

In addition, HCWs should be encouraged to keep up-to-date with recent scientific literature and advances in TB management and control as well as being aware of national and international guidelines and best practices [23]. In such regards, it is disappointing that less than a quarter of HCWs in our study reported attending a lecture / seminar / workshop on TB in the previous year which may also explain the lack of knowledge regarding recent diagnosis techniques such as GeneXpert MTB/RIF in our study. While appropriate training and education of HCWs is crucial to improving their KAP regarding TB, such activities alone may not be sufficient for improving HCWs performance as well as the quality of TB control outcomes [42]. Therefore, special attention should also be given to addressing factors beyond training such as lifestyle, health system, job satisfaction as well as Hajj-related factors, that could influence HCWs’ KAP regarding TB, so as not to reinforce the limitations and weaknesses of current practices. The above factors are part of a “productivity mix” that impact on HCWs’ job motivation, productivity and performance, which may translate to HCWs not providing care according to standards and not being responsive to the needs of patients.[44] A number of strategies to improve productivity, responsiveness and competences of HCWs can be implemented at both health-system and heath facility levels. These may include changes in payment systems and accountability mechanisms, supportive supervision as well as quality assurance and performance-improvement including performance-based incentives.[44, 45]

Our study has some limitations. While we enrolled a large number of HCWs of different occupations from numerous healthcare facilities in Hajj, the sample size however represents a small proportion of the thousands of HCWs deployed to work in Hajj each year. The latter, in addition to the cross-sectional design of the study, limits the generalizability of the findings. Also, we collected information using a questionnaire, therefore, responses obtained were prone to information bias. Similarly, we did not measure actual practice by observing staff behaviour in a specific situation or by using the simulated client method, hence, there may have been an overestimation of good practice among the HCWs.

Notwithstanding the above limitations, this is the first attempt to study KAP of HCWs deployed during Hajj regarding TB. The identified knowledge gaps and poor attitudes and practices can serve as baseline data to design effective interventions to address these shortcomings. Further studies at larger scales including qualitative methods and observation of actual practices as well as considering availability of resources and other possible infrastructure constrains are warranted.

## Supporting information

S1 Table Summary statistics of knowledge questions regarding TB among HCWs. (DOCX)

S1 File Tuberculosis knowledge, attitude and practice among healthcare workers during the 2016 Hajj- Study questionnaire. (PDF)
