# Supplementary material for: Tuberculosis knowledge, attitude and practice among healthcare workers during the 2016 Hajj
Source: PLoS One. 2019 Jan 25;14(1):e0210913. doi: 10.1371/journal.pone.0210913 (PMC6347151; doi:10.1371/journal.pone.0210913)
Supplement: S1 Table — (DOCX) [file pone.0210913.s001.docx]

**S1 Table. Summary statistics of knowledge questions regarding TB among HCWs**

| **Knowledge question** | **Category** | **Freq** | **%** |
| --- | --- | --- | --- |
| **1. TB falls under** |  |  |  |
|  | Viral infection | 101 | 20.82 |
|  | Bacterial infection | 364 | 75.05 |
|  | Don't know | 19 | 3.92 |
|  | Both | 1 | 0.21 |
| **2. PTB main symptoms** |  |  |  |
|  | Don't know | 11 | 2.07 |
|  | Answered | 520 | 97.93 |
|  |  |  |  |
|  | Cough ≥3 weeks | 412 | 79.23 |
|  | Fever/chills | 328 | 63.08 |
|  | Dizziness | 49 | 9.42 |
|  | Tiredness/ fatigue | 243 | 46.73 |
|  | Cough with blood | 393 | 75.58 |
|  | Weight loss | 371 | 71.35 |
|  | Headache | 84 | 16.15 |
|  | Pain with urination | 10 | 1.92 |
|  | Diarrhoea | 48 | 9.23 |
|  | Chest pain | 209 | 40.19 |
|  | Loss of appetite | 295 | 56.73 |
|  | Memory loss | 7 | 1.35 |
|  | Night sweats | 274 | 52.69 |
|  | Blurry vision | 11 | 2.12 |
| **3. PTB diagnostic tests** |  |  |  |
|  | Don't know | 14 | 2.63 |
|  | Answered | 519 | 97.37 |
|  |  |  |  |
|  | TST | 341 | 65.70 |
|  | Sputum culture | 360 | 69.36 |
|  | Urine examination | 15 | 2.89 |
|  | GeneXpert MTB/RIF | 62 | 11.95 |
|  | Sputum AFB smear | 299 | 57.61 |
|  | IGRA | 69 | 13.29 |
|  | Liver function | 43 | 8.29 |
|  | CBC | 68 | 13.10 |
|  | Blood culture | 61 | 11.75 |
|  | Chest X-ray | 370 | 71.29 |
| **4. PTB transmission** |  |  |  |
|  | Answered | 529 | 97.9 |
|  |  |  |  |
|  | Blood transmission | 51 | 9.64 |
|  | Coughing | 492 | 93.01 |
|  | Sharing food or drink | 143 | 27.03 |
|  | Contaminated surfaces | 221 | 41.78 |
|  | Sneezing | 344 | 65.03 |
|  | Kissing | 195 | 36.86 |
|  | Shaking hand | 89 | 16.82 |
| **5. Latent TB screening tests** |  |  |  |
|  | Don't know | 38 | 7.24 |
|  | Answered | 487 | 92.76 |
|  |  |  |  |
|  | TST | 292 | 59.96 |
|  | Sputum culture | 216 | 44.35 |
|  | Urine examination | 11 | 2.26 |
|  | GeneXpert MTB/RIF | 35 | 7.19 |
|  | Sputum AFB smear | 172 | 35.32 |
|  | IGRA | 62 | 12.73 |
|  | Liver function | 25 | 5.13 |
|  | CBC | 38 | 7.80 |
|  | Blood culture | 30 | 6.16 |
|  | Chest X-ray | 258 | 52.98 |
| **6.Percentage of active PTB positive for TB on smear microscopy** |  |  |  |
|  | 10% | 24 | 4.40 |
|  | 20% | 24 | 4.40 |
|  | 50% | 88 | 16.3 |
|  | 80% | 112 | 20.7 |
|  | Don’t know | 292 | 54.1 |
| **7. Most useful sputum collection method for PTB diagnosis** |  |  |  |
|  | Don't know | 88 | 16.83 |
|  | Answered | 436 | 83.17 |
|  |  |  |  |
|  | 3 samples (spot-morning-spot) | 380 | 87.36 |
|  | 2 spot, 1 spot samples | 28 | 6.44 |
|  | 1 spot sample | 18 | 4.14 |
|  | 2 spot samples | 10 | 2.30 |
| **8. 1^st^-line anti-TB drugs** |  |  |  |
|  | Don't know | 145 | 27.57 |
|  | Answered | 381 | 72.43 |
|  |  |  |  |
|  | INH | 299 | 78.48 |
|  | RIF | 318 | 83.46 |
|  | CIP | 36 | 9.45 |
|  | LEV | 8 | 2.10 |
|  | CAP | 5 | 1.31 |
|  | KAN | 14 | 3.67 |
|  | EMB | 221 | 58.01 |
|  | PZA | 209 | 54.86 |
|  | AMK | 12 | 3.15 |
| **9. 2^nd^-line anti-TB drugs** |  |  |  |
|  | Don't know | 197 | 39.01 |
|  | Answered | 308 | 60.99 |
|  |  |  |  |
|  | INH | 96 | 31.17 |
|  | RIF | 93 | 30.19 |
|  | CIP | 100 | 32.47 |
|  | LEV | 51 | 16.56 |
|  | CAP | 49 | 15.91 |
|  | KAN | 82 | 26.62 |
|  | EMB | 87 | 28.25 |
|  | PZA | 85 | 27.60 |
|  | AMK | 84 | 27.27 |
| **10. MDR-TB bacteria is resistant to** |  |  |  |
|  | INH | 19 | 3.79 |
|  | RIF | 12 | 2.40 |
|  | At least INH & RIF | 125 | 24.95 |
|  | EMB | 8 | 1.60 |
|  | At least CIP & KAN | 13 | 2.59 |
|  | All of the above | 106 | 21.16 |
|  | Don't know | 206 | 41.12 |
|  | Any combinations of the above | 12 | 2.40 |
| **11. XDR-TB is** |  |  |  |
|  | Don't know | 259 | 54.07 |
|  | MDR-TB resistant to any fluoroquinolone and at least one of three injectable 2^nd^-line drugs | 61 | 12.73 |
|  | TB resistant to any fluoroquinolone and at least one of three injectable 2^nd^-line drugs | 54 | 11.27 |
|  | TB resistant to all 1^st^-line anti-TB drugs | 101 | 21.09 |
|  | Any combinations of the above | 4 | 0.84 |
| **12. Length of standard treatment of drug-sensitive TB** |  |  |  |
|  | Don't know | 108 | 21.05 |
|  | 1-3 Months | 95 | 18.52 |
|  | 4-6 Months | 122 | 23.78 |
|  | 6-9 Months | 159 | 30.99 |
|  | >12 Months | 26 | 5.07 |
|  | Any combinations of the above | 3 | 0.58 |
| **13. HIV patients are more vulnerable to contracting TB** |  |  |  |
|  | Don't know | 62 | 11.95 |
|  | True | 422 | 81.31 |
|  | False | 35 | 6.74 |
| **14. PTB is curable** |  |  |  |
|  | Don't know | 39 | 7.54 |
|  | True | 456 | 88.2 |
|  | False | 22 | 4.26 |
| **15. MDR-TB is curable** |  |  |  |
|  | Don't know | 161 | 31.94 |
|  | True | 256 | 50.79 |
|  | False | 87 | 17.26 |
| **16. BCG vaccination is protective against TB** |  |  |  |
|  | Don't know | 140 | 27.24 |
|  | True | 189 | 36.77 |
|  | False | 185 | 35.99 |
| **17. PTB is transmitted via the airborne route** |  |  |  |
|  | Don't know | 39 | 7.69 |
|  | True | 424 | 83.63 |
|  | False | 44 | 8.68 |
| **18. Patients with latent TB can spread the disease** |  |  |  |
|  | Don't know | 62 | 12.04 |
|  | True | 284 | 55.15 |
|  | False | 169 | 32.82 |
| **19. Patients with latent TB have positive reaction on TST/IGRA tests** |  |  |  |
|  | Don't know | 160 | 31.68 |
|  | True | 290 | 57.43 |
|  | False | 55 | 10.89 |
| **20. Patient with TB become non-infectious soon after initiating appropriate treatment** |  |  |  |
|  | Don't know | 76 | 14.84 |
|  | True | 208 | 40.63 |
|  | False | 228 | 44.53 |
| **21. Only patients with active TB can spread the disease** |  |  |  |
|  | Don't know | 63 | 12.33 |
|  | True | 306 | 59.88 |
|  | False | 142 | 27.79 |
| **22. Appropriate PPE to use with active PTB patients** |  |  |  |
|  | Don't know | 25 | 4.86 |
|  | Surgical mask | 92 | 17.9 |
|  | N95 respirator | 393 | 76.46 |
|  | No PPE is needed | 4 | 0.78 |
| **23. Using PPE to protect HCWs from TB is important** |  |  |  |
|  | Don’t know | 29 | 5.56 |
|  | Yes | 469 | 89.85 |
|  | No | 24 | 4.60 |

TB; tuberculosis, PTB; pulmonary tuberculosis, HIV; human immunodeficiency virus, PPE; personal protective equipment, HCW; healthcare worker, MDR; multidrug-resistant, XDR; extensively drug-resistant, TST: tuberculin skin tests (TST), AFB; acid-fast bacilli, IGRA; interferon gamma release assay, CBC; complete blood count, INH; Isoniazid, RIF; Rifampicin, CIP; Ciprofloxacin, PZA; Pyrazinamide, LEV; Flevofloxacin, KAN; Kanamycin, AMK; Amikacin, EMB; Ethambutol , CAP; Capreomycin, BCG; Bacillus Calmette-Guérin
