# Supplementary material for: Tuberculosis knowledge, attitude and practice among healthcare workers during the 2016 Hajj
Source: PLoS One. 2019 Jan 25;14(1):e0210913. doi: 10.1371/journal.pone.0210913 (PMC6347151; doi:10.1371/journal.pone.0210913)
Supplement: S1 File — (PDF) [file pone.0210913.s002.pdf]

Tuberculosis knowledge, attitude and practice among healthcare workers during the 2016 Hajj- Study questionnaire

Section (1) : Healthcare worker (HCW) information

1.1. Gender :
- ☐ Male
- ☐ Female

1.2. Age (Years): ____________________

1.3. Nationality : ____________________

1.4. Highest level of education:
- ☐ Diploma
- ☐ Bachelor
- ☐ Master
- ☐ Doctorate/Speciality certificate
- ☐ **Other Please Specify** ____________________

1.5. Occupation:
- ☐ Physician : **(Please Specify**)
  - ☐ Resident
  - ☐ Specialist
  - ☐ Consultant
  - ☐ General practitioner
  - ☐ **Other (please specify)** ____________________
- ☐ Nurse : **(Please Specify**)
  - ☐ Infection control nurse
  - ☐ **Other (please specify)** ____________________

Other :
- ☐ Laboratory / Diagnostics
- ☐ Pharmacist
- ☐ **Other (please specify)** ____________________

1.6. Ward stationed in during your non-Hajj health facility (Tick all that apply) :
- ☐ ICU
- ☐ Surgical ward
- ☐ ER
- ☐ Pediatric ward
- ☐ Medical ward
- ☐ Maternity (O&G) ward
- ☐ Isolation ward
- ☐ **Other (please specify)** ____________________

1.7. How long have you been working in this current position?
- ☐ <1 year
- ☐ >5-10 years
- ☐ 1-5 years
- ☐ >10 years

1.8. Do you deal with pulmonary TB patients in your non-Hajj health facility?
- ☐ Yes
- ☐ No

1.9. Ward stationed in during Hajj :
- ☐ ICU
- ☐ ER
- ☐ Medical ward
- ☐ Surgical ward
- ☐ Pediatric ward
- ☐ Maternity (O&G) ward
- ☐ **Other (please specify)** ____________________

1.10. During your Hajj assignment, did you deal with pulmonary TB cases?
- ☐ Yes
- ☐ No

Section (2) : TB Knowledge

2.1. In the past 12 months, have you attended a lecture / seminar / workshop on TB?
- ☐ Yes
- ☐ No
- ☐ Don't know

2.2. TB falls under:
- ☐ Viral infection
- ☐ Bacterial Infection
- ☐ Don't know

2.3. Which of these is/are main symptom(s) of pulmonary TB? (Tick all that apply)
- ☐ Cough ≥3 weeks
- ☐ Pain with urination
- ☐ Fever/chills
- ☐ Diarrhea
- ☐ Dizziness
- ☐ Chest pain
- ☐ Tiredness/fatigue
- ☐ Loss of appetite
- ☐ Cough with blood
- ☐ Memory loss
- ☐ Weight loss
- ☐ Night sweats
- ☐ Headache
- ☐ Blurry vision
- ☐ Don't know

2.4. Which of the following is/are diagnostic test(s) for pulmonary TB? (Tick all that apply) :
- ☐ Mantoux tuberculin skin tests (TST)
- ☐ Liver function test
- ☐ Sputum culture
- ☐ Complete Blood Count (CBC)
- ☐ Urine examination
- ☐ Blood Culture
- ☐ GeneXpert MTB/RIF test
- ☐ Chest x-ray
- ☐ Sputum Acid-fast bacilli (AFB) smear
- ☐ Don't know
- ☐ Interferon Gamma Release Assay (IGRA)

2.5. Which of the following is/are the mode of pulmonary TB transmission? (Tick all that apply)
- ☐ Blood transmission
- ☐ Sneezing
- ☐ Coughing
- ☐ Kissing
- ☐ Sharing food or drink
- ☐ Shaking hand
- ☐ Touching surfaces contaminated with *M. tuberculosis*

2.6. Which of the following is/are screening test(s) for latent TB infection? (Tick all that apply)
- ☐ Mantoux tuberculin skin tests (TST)
- ☐ Liver function test
- ☐ Complete Blood Count (CBC)
- ☐ Sputum culture
- ☐ Urine examination
- ☐ Blood Culture
- ☐ GeneXpert MTB/RIF test
- ☐ Chest x-ray
- ☐ Sputum Acid-fast bacilli (AFB) smear
- ☐ Don't know
- ☐ Interferon Gamma Release Assay (IGRA)

2.7. What percentage of active pulmonary TB will be positive for TB on smear microscopy :
- ☐ 10%
- ☐ 50%
- ☐ 20%
- ☐ 80%
- ☐ Don't know

Tuberculosis knowledge, attitude and practice among healthcare workers during the 2016 Hajj- Study questionnaire

2.8. What method of sputum sample collection is the most useful for pulmonary TB diagnosis?
- ☐ 3 (spot, morning, spot) samples
- ☐ 1 spot sample
- ☐ 2 spot, 1 spot samples
- ☐ 2 spot samples
- ☐ Don't know

2.9. Which of these is/are first line anti-TB drugs: (Tick all that apply)
- ☐ Isoniazid (INH)
- ☐ Kanamycin (KAN)
- ☐ Rifampicin (RIF)
- ☐ Ethambutol (EMB)
- ☐ Ciprofloxacin (CIP)
- ☐ Pyrazinamide (PZA)
- ☐ Flevofloxacin (LEV)
- ☐ Amikacin (AMK)
- ☐ Capreomycin (CAP)
- ☐ Don’t know

2.10. Which of these is/are a second-line anti-TB drugs: (Tick all that apply)
- ☐ Isoniazid (INH)
- ☐ Kanamycin (KAN)
- ☐ Rifampicin (RIF)
- ☐ Ethambutol (EMB)
- ☐ Ciprofloxacin (CIP)
- ☐ Pyrazinamide (PZA)
- ☐ Flevofloxacin (LEV)
- ☐ Amikacin (AMK)
- ☐ Capreomycin (CAP)
- ☐ Don’t know

2.11. Multidrug-resistant tuberculosis (MDR-TB) is caused by bacteria resistant to:
- ☐ Isoniazid
- ☐ Rifampicin
- ☐ at least Isoniazid and Rifampicin
- ☐ Ethambutol
- ☐ at least Ciprofloxacin and Kanamycin
- ☐ Don't know
- ☐ All of the above

2.12. Extensively drug-resistant TB (XDR-TB) is:
- ☐ MDR-TB resistant to any fluoroquinolone and at least one of three injectable second-line drugs
- ☐ TB resistant to any fluoroquinolone and at least one of three injectable second-line drugs
- ☐ TB resistant to all first-line anti-TB drug
- ☐ Don't know

2.13. The standard treatment for new patient with drug-sensitive TB is:
- ☐ 1-3 Months
- ☐ 4-<6 Months
- ☐ 6-9 Months
- ☐ >12 Months
- ☐ Don't know

2.14. HIV patient are more vulnerable to contracting TB :
☐ True ☐ False ☐ Don't Know

2.15. Pulmonary TB is a curable disease :
☐ True ☐ False ☐ Don't Know

2.16. MDR-TB is a curable disease :
☐ True ☐ False ☐ Don't Know

2.17. People that have received bacilli Calmette-Guerin (BCG) vaccination do not develop active TB :
☐ True ☐ False ☐ Don't Know

2.18. Airborne is a mode of transmission of pulmonary TB:
☐ True ☐ False ☐ Don't Know

2.19. Patient with latent TB can spread the disease:
☐ True ☐ False ☐ Don't Know

2.20. Patient with latent TB have a positive reaction to TST or IGRA:
☐ True ☐ False ☐ Don't Know

2.21. Patient with TB usually become noninfectious soon after initiating appropriate treatment:
☐ True ☐ False ☐ Don't Know

2.22. Only patient with active TB can spread the disease:
☐ True ☐ False ☐ Don't Know

2.23. Which Personal Protective Equimenent (PPE) you should use when dealing with a patient with active pulmonary TB:
- ☐ Surgical mask
- ☐ N95 respirator
- ☐ No PPE is needed
- ☐ Don't know

2.24. Using PPE to protect HCWs from TB is important:
☐ Yes ☐ No ☐ Don’t know

Section (3): Attitude towards TB/TB patients

3.1.Would you be willing to work in a TB clinic/ward?
☐ Yes ☐ No

3.2.Would you resign from work if you are posted to a TB clinic/ward?
☐ Yes ☐ No

3.3.Would you be willing to be screened for TB if you had suggestive symptoms?
☐ Yes ☐ No

3.4.Would you be willing to teach patients and co-workers about TB prevention?
☐ Yes ☐ No

3.5. Do you think all TB patients should be isolated for treatment?

☐ Yes ☐ No ☐ Don’t know

3.6. It is ok to allow a TB patient to leave the hospital soon after initiating appropriate treatment:

☐ Yes ☐ No ☐ Don’t know

3.7. Would you be willing to attend seminars on TB:

☐ Yes ☐ No

3.8. Would you recommend the suspension of treatment if a TB patient is feeling better?

☐ Yes ☐ No ☐ Not applicable

3.9. Would you start TB treatment for a TB patient before diagnosis is confirmed if a suspected TB patient is very ill?

☐ Yes ☐ No ☐ Not applicable

3.10. Would you use a face mask when dealing with a pulmonary TB patient even when it is uncomfortable:

☐ Yes ☐ No

3.11. Would you be willing to teach patient how to collect sputum sample:

☐ Yes ☐ No ☐ Not applicable

3.12. Would you trust the result the laboratory provides you on sputum cultures:

☐ Yes ☐ No ☐ Not applicable

3.13. I would not accept to examine/treat a TB patients:

☐ Yes ☐ No

3.14. I have a very low risk of acquiring TB from my patients:

☐ Yes ☐ No ☐ Don’t know

3.15. I worry about acquiring active TB disease while at work:

☐ Yes ☐ No

Section (4): TB Practice

4.1. I usually perform hand hygiene and wear PPE before contact with pulmonary TB patient/TB samples:

☐ Yes ☐ No ☐ Not applicable

4.2. I usually wear N95 respirator when caring for patient with pulmonary TB/working on TB samples:

☐ Yes ☐ No ☐ Not applicable

4.3. I request sputum tests when I suspect active TB:

☐ Yes ☐ No ☐ Not applicable

4.4. Always put the patient with active TB in an isolated room:

☐ Yes ☐ No ☐ Not applicable

4.5. I open windows when possible in TB patients rooms to increase natural ventilation:

☐ Yes ☐ No ☐ Not applicable

4.6. I order HIV test when I diagnose active TB:

☐ Yes ☐ No ☐ Not applicable

4.7. Always put the patient with known TB separated from HIV patients:

☐ Yes ☐ No ☐ Not applicable

4.8. Sometimes I use wet or soiled N95 respirator:

☐ Yes ☐ No ☐ Not applicable

4.9. I always make sure that samples are sputum and not saliva before sending them to laboratory/before testing in the laboratory:

☐ Yes ☐ No ☐ Not applicable

4.10. I commence anti-TB drugs on suspect TB cases before lab confirmation:

☐ Yes ☐ No ☐ Not applicable

4.11. I request contact tracing for all confirmed TB cases:

☐ Yes ☐ No ☐ Not applicable

4.12. I request liver function tests before starting anti-TB treatment:

☐ Yes ☐ No ☐ Not applicable

4.13. I start contacts of active TB cases who are positive for IGRA/TST tests on Isoniazid /Rifampicin prophylaxis:

☐ Yes ☐ No ☐ Not applicable
